# Teaching old drugs new tricks to stop malaria invasion in its tracks

**DOI:** 10.1186/s12915-015-0185-6

**Published:** 2015-09-08

**Authors:** Vasant Muralidharan, Boris Striepen

**Affiliations:** Center for Tropical and Emerging Global Diseases and Department of Cellular Biology, University of Georgia, Athens, GA 30602 USA

## Abstract

Malaria is a common and life-threatening disease endemic in large parts of the world. The emergence of antimalarial drug resistance is threatening disease-control measures that depend heavily on treatment of clinical malaria. The intracellular malaria parasite is particularly vulnerable during its brief extracellular stage of the life cycle. Wilson et al. describe a screen targeting these extracellular parasite stages and make the surprising discovery that clinically used macrolide antibiotics are potent inhibitors of parasite invasion into erythrocytes.

See research article: http://www.biomedcentral.com/1741-7007/13/52

## Commentary

Malaria is a dangerous and common disease that stalks most of the tropical and sub-tropical parts of the world, home to about 3.5 billion people. The disease is caused by single-celled obligate intracellular eukaryotic parasites of the genus *Plasmodium* and is associated with significant mortality in the case of one species, *Plasmodium falciparum.* The lifecycle of *Plasmodium* is complicated, with sexual growth within mosquitos and asexual growth within vertebrate hosts, including humans. Transmission depends upon the bite of an infected mosquito. Thus, control of malaria relies in part on insecticide-treated bed nets to prevent mosquitos from feeding. Another mainstay of malaria control are antimalarials that treat clinical disease and are used in preventative or targeted mass drug administration for specific populations. These disease control mechanisms have resulted in a significant reduction in the burden of malaria. Over the past decade, mortality from malaria has been reduced to 600,000 deaths per year from a peak of nearly 1.1 million deaths per year [1], a 45 % decrease attributed to the use of bed nets and to the antimalarial drug artemisinin [1].

## Resistance at the front line

Artemisinin combination therapies, which are combinations of potent but short-lived artemisinin with long acting partners, have been very successful in combating malaria. However, this progress is under grave threat because of emergence of artemisinin-resistant *P. falciparum* [2]. Clinical resistance to artemisinin is not readily spotted in short-term parasite growth assays, but reveals itself in the peculiar ability of the parasites to ‘hibernate’ in the presence of drug, ready to rebound as soon as treatment is discontinued. The hallmark of resistance is a reduced rate of parasite clearance. Mutations in the K13 kelch propeller domain protein have been associated with this phenotype through genetic analysis of resistant parasites selected deliberately in the laboratory and collected from natural infection in the field [3]. While clearly associated with the mechanism of resistance, this protein is likely not the direct molecular target of the drug. Proteins that associate with the kelch protein are among the candidates [4], but the case is far from closed at this point. Regardless of mechanism, to preserve the gains against clinical malaria in the face of the parasite’s remarkable ability to develop drug resistance it is essential that we keep step with a deep portfolio of new drugs ready to take over when inevitable resistance breaks through.

## Common antibiotics as invasion-inhibitory anti-malarials

In research recently reported in *BMC Biology* Wilson and colleagues [5] seek to add to the anti-malarial portfolio with a screen for inhibitors of parasite host cell invasion. Surprisingly, among the best compounds to emerge from this effort are well known macrolide antibiotics, including azithromycin, erythrocymicin, and roxithromycin, that were found to inhibit invasion of red blood cells by the malaria parasite [5]. Azithromycin and its analogues had the most potent effect.

The use of antibiotics is not new in the treatment of malaria [6]; in particular, inhibitors of bacterial protein translation are known to kill *P. falciparum*. This initially may be unexpected for a eukaryotic pathogen, but the discovery of the parasite plastid or apicoplast revealed a possible target [7]. Like all plastids, this parasite organelle evolved form cyanobacterial ancestors and is susceptible to inhibition of its prokaryotic ribosomes [8]. The apicoplast is an essential organelle required for the production of several important metabolites [9]. However, inhibition of protein translation in the apicoplast leads to a ‘delayed death’ [8]. This peculiar feature of antibiotic action on parasite growth and the resulting lag phase in the onset of efficacy may limit the usefulness of these drugs against acute infection.

**Fig. 1.**
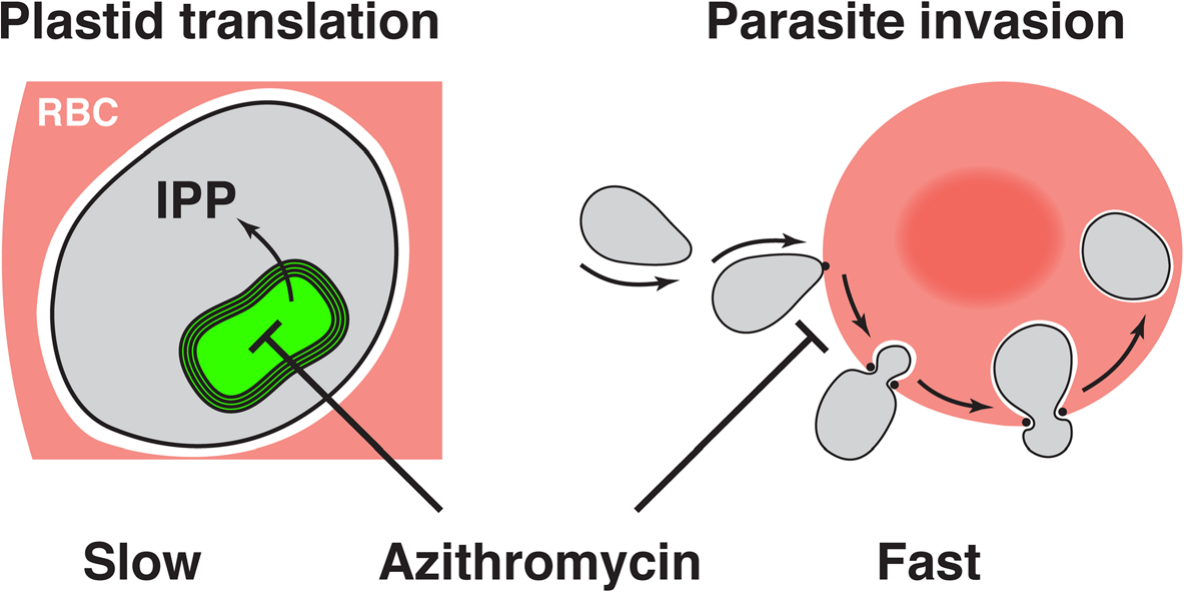
Two independent targets for macrolide antibiotics in *Plasmodium falciparum*. Azithromycin inhibits protein synthesis in the apicoplast (*green*). Loss of translation in the plastid ultimately starves the parasite (*grey*) for the essential isoprenoid precursor isopentenyl-pyrophosphate (*IPP*). Wilson et al. describe a second mode of action in which azithromycin blocks an early step in the process used by the parasite to invade red blood cells (*RBC*, *red*). This effect is much faster, but requires higher concentrations of drug

So did Wilson *et al*. rediscover plastid inhibitory activity of macrolides or are they on the trail of something new? In a set of time-limited drug exposure experiments they convincingly demonstrate a fast invasion inhibitory activity that is independent of and in addition to the slow plastid effect of azithromycin (Fig. 1) [5]. Observing this phenomenon was made possible by a recently developed method for isolation of viable invasive stages of *P. falciparum* [10]. Incubating purified invasive stages with red blood cells for short periods followed by drug washout resulted in almost complete loss of parasite invasion [5]. On the other hand, similar incubation of post-invasion life stages with azithromycin had no effect on parasite growth [5]. The two-target hypothesis was also supported by medicinal chemistry and structure function analysis: some macrolide analogues showed increased activity against merozoite invasion while their anti-apicoplast activity remained unchanged from that of azithromycin [5].

## The promise and problems of antibiotics as dual action anti-malarials

A fast acting antibiotic could be very attractive for malaria therapy. Antibiotics are well-worn tools of the medical trade and their established clinical profile, good safety record, and moderate cost could fast track new treatments. Relatively high concentrations of azithromycin are required to block invasion, but these may not be as easily or safely reached *in vivo* [5]. Conceptually, focusing on invasion narrows the opportunity for chemical interference to a very important but also very brief time period. Invasion takes place in about 120 seconds of the 48 hour growth cycle. Lastly, *Plasmodium* and the related apicomplexan parasite *Toxoplasma gondii* have demonstrated significant flexibility and quickly adapt to experimental insults directed at their invasion machinery [11]. This includes the genetic deletion or chemical removal of ligands and adapters from the parasite and the host, revealing a buffer of redundancy around the essential event of invasion. Insight into the mechanism of action and the potential redundancy of the specific molecular target will be crucial to understand whether azithromycin is a bullet that parasite invasion ultimately can or cannot dodge.

## How do antibiotics block invasion?

Forward genetics would be the weapon of choice to attack the mode of action of the antibiotic invasion block. The malaria parasite is haploid and isolation of resistance mutants followed by genetic mapping has been a highly successful way to define drug targets [12]. In a decade long campaign, the Wellems laboratory at the NIH pioneered this genetic mapping method to discover the mutations responsible for chloroquine resistance. The advent of low cost whole genome sequencing has been truly transformative, yielding high-density single nucleotide polymorphism maps to compare sensitive and resistant lines with reasonable investment. At the same time, transfection experiments to directly test whether a mutation is the cause of resistance have become more and more powerful. CRISPR/Cas9 systems now allow marker-free genome editing to rigorously validate such mutations. Unfortunately, the dual-mode of action of azithromycin makes the isolation and analysis of invasion-specific azithromycin resistance mutants a non-trivial undertaking.

The target of azithromycin appears broadly conserved as the drug also inhibits invasion of other apicomplexan species into their respective host cells [5]. Azithromycin-treated parasites bind to but then let go of their host without productive invasion. In the presence of drug, they appear unable to form a moving junction, a unique parasite induced structure linking host and parasite membranes and used by the parasite to propel itself into the red blood cell. There are several possible ways by which azithromycin might inhibit parasite invasion, an essential process required for parasite propagation and spread. Azithromycin may interfere with vital ligand-receptor interactions. Disruption of parasite invasion in an analogous fashion by targeting the host protein basigin with recombinant chimeric antibodies cures established infections in a humanized mouse model [13]. As invasion relies on secretion and delivery of parasite factors to the host this may be another potential target. Interference with the complex regulatory machinery of the invasion process provides additional candidates. A new stem cell-derived model to dissect *Plasmodium* invasion opens the door to manipulation of both host and parasite components at a level previously not attainable [14]. By the same token, using azithromycin as a chemical biology tool compound could prove to be a highly complementary approach to understand not only its mode of action but the invasion process in general.

Overall, this study should reignite interest in antibiotics as anti-malaria drugs and specifically spur future studies into macrolides and invasion. Utilizing these antibiotics in combination with artemisinin may even slow the spread of artemisinin resistance — and in this context their dual activity may be an asset. Since macrolides are already in clinical use, there is a wealth of information and experience in their use, adding to the list of urgently needed drug candidates at a time when the frontline antimalarial, artemisinin, is losing its efficacy.
